# Analysis of the Risk Factors for Mortality in Adult COVID-19 Patients in Wuhan: A Multicenter Study

**DOI:** 10.3389/fmed.2020.00545

**Published:** 2020-08-25

**Authors:** Man Li, Biao Cheng, Wen Zeng, Sichao Chen, Mengqi Tu, Meng Wu, Wei Tong, Shipei Wang, Yihui Huang, Wei Long, Wei Zhou, Danyang Chen, Lin Zhou, Min Wang, Haibo Xu, Aiping Deng, Zeming Liu, Liang Guo

**Affiliations:** ^1^Department of Plastic Surgery, Zhongnan Hospital of Wuhan University, Wuhan, China; ^2^Department of Pharmacy, Tongji Medical College, The Central Hospital of Wuhan, Huazhong University of Science and Technology, Wuhan, China; ^3^Department of Ophthalmology, Zhongnan Hospital of Wuhan University, Wuhan, China; ^4^Department of Radiology, Zhongnan Hospital of Wuhan University, Wuhan, China; ^5^Department of Ultrasound, Zhongnan Hospital of Wuhan University, Wuhan, China; ^6^Department of Orthopedics, Tongji Medical College, Union Hospital, Huazhong University of Science and Technology, Wuhan, China

**Keywords:** COVID-19, mortality, risk factors, SARS-CoV-2, Wuhan

## Abstract

**Objectives:** An outbreak of coronavirus disease (COVID-19) in 2019 in Wuhan, China, has spread quickly worldwide. However, the risk factors associated with COVID-19-related mortality remain controversial.

**Methods:** A total of 245 adult patients with laboratory-confirmed COVID-19 from two centers were analyzed. Chi-square, Fisher's exact, and the Mann-Whitney *U*-tests were used to compare the clinical characteristics between the survivors and non-survivors. To explore the risk factors associated with in-hospital death, univariable and multivariable cox regression analyses were used.

**Results:** Of the 245 patients included in this study, 23 (9.4%) died in the hospital. The multivariate regression analysis showed increased odds of in-hospital deaths associated with age, D-dimer levels >1,000 ng/L, platelet count <125, and higher serum creatinine levels.

**Conclusions:** We identified risk factors that show significant association with mortality in adult COVID-19 patients, and our findings provide valuable references for clinicians to identify high-risk patients with COVID-19 at an early stage.

## Introduction

In December 2019, infectious pneumonia broke out in Wuhan, Hubei province. It was caused by a new coronavirus, which was named “SARS-CoV-2” by the World Health Organization (WHO) on February 13, 2020 (1). Meanwhile, the infectious disease caused by SARS-CoV-2 was named “COVID-19.” In China, according to the National Health Commission (2), a total of 82,341 cases were diagnosed, of which 77,892 patients were discharged, and 3,342 died as of April 15. Since the early stages of the outbreak, the disease has spread to most countries in the world. As of April 15, 2020, more than 1,900,000 people have been infected and over 120,000 people have died worldwide (3). The situation is likely to worsen with the rapid escalation and global spread of infection (4).

The symptoms for COVID-19 vary from cough and fever to dyspnea, which can be difficult to identify. Approximately 20% of the patients are estimated to develop severe illness, with overall mortality around 2.3% (5). Given that there are no drugs that have been proven to be clinically effective in targeting the SARS-CoV-2 directly, it is particularly important to identify the risk factors associated with disease progression and mortality (6, 7).

In this study, we explored the potential host risk factors associated with death in a retrospective cohort of 245 laboratory-confirmed COVID-19 patients admitted to the two appointed hospitals in Wuhan. We present a detailed review of the medical information of each patient to clarify the clinical manifestations, laboratory test results, and outcomes to better understand the disease progression and prognosis.

## Materials and Methods

### Study Design and Patients

This multiple center retrospective study was approved by the Institutional Ethics Board of the Zhongnan Hospital of Wuhan University (NO. 2020015) and the Central Hospital of Wuhan (NO. 2020072). Oral informed consent was obtained from patients before data collection with their privacy rights protected. According to the Seventh Interim Guidance of Diagnosis and Treatment of COVID-19 published by the Chinese National Health Commission on March 3, 2020 (8), patients with radiological characteristics of viral pneumonia were recognized as suspected cases. Meanwhile, patients who tested positive with the real-time reverse-transcription–polymerase-chain-reaction (RT-PCR) test for SARS-CoV-2 or showed high-level homology with SARS-CoV-2 detected through gene sequencing were diagnosed as confirmed cases. Therefore, patients who were negative on repeated RT-PCR test and those without clear outcomes within the observation cut-off time were excluded from the study. Ultimately, we enrolled a total of 245 patients who were admitted to Zhongnan Hospital of Wuhan University and the Central Hospital of Wuhan between December 26, 2019, and February 15, 2020. All the patients were followed up until March 7, 2020.

### Data Collection

We collected data on the demographic, clinical, laboratory, and radiological characteristics, as well as treatment and clinical outcomes of the included patients. All the information was acquired from their electronic records and independently checked by three participants (SW, SC, and YH) to verify data accuracy.

The demographic features included gender (male or female), age at diagnosis, comorbidities, symptoms, and time from onset to admission. Laboratory tests included routine blood tests, blood coagulation tests, and tests for renal and liver functions, creatine kinase, infection-related biomarkers, brain natriuretic peptide (BNP), and RT-PCR analysis for SARS-CoV-2. The radiographic examination included chest CT or X-ray. Clinical management and outcomes data included information on therapies, and clinical outcomes until the end of the follow-up period, time from onset to a negative RT-PCR result, and changes in chest x-rays and CT scans.

### Statistical Analysis

The baseline characteristics of survivors and non-survivors were compared. Categorical and continuous variables were presented as n (%) and median (IQR), respectively. The Chi-square test or Fisher's exact test were performed for categorical variables, and the Mann-Whitney *U*-test was used for continuous variables. Univariable and multivariable cox regression analyses were applied to investigate the risk factors associated with in-hospital deaths. Candidate factors for the multivariable analysis were chosen on the basis of the previous findings and findings of the univariable analysis. For laboratory results, we considered the normal ranges used in the Zhongnan Hospital of Wuhan University as the reference. All data analyses were performed using SPSS (version 26.0).

## Results

### Demographics and Clinical Characteristics of Patients With COVID-19

The 245 laboratory-confirmed COVID-19 patients were divided into survivor (*n* = 222) and non-survivor (*n* = 23) groups. The demographic characteristics and symptoms of all the patients are shown in Table 1. The median age of all patients was 54 [interquartile range (IQR): 37–64] years, while that of patients in the survivor group was 52 years. The median age of the non-survivor group was 76 years. The proportion of men (48.2%) and women (51.8%) was comparable across the whole cohort. Female patients accounted for a higher percentage (55.0%) of the survivor group, while male patients accounted for the majority (78.3%) of the non-survivor group. About half the patients (*n* = 122) had underlying diseases including hypertension, diabetes, chronic obstructive lung disease, cardiovascular disease (CVD), carcinoma, nervous system disease, and hepatic disease. Hypertension was the most common comorbidity in the non-survivor group, followed by CVD. The most common symptoms on admission were fever and cough, followed by fatigue and expectoration (Table 1). The median time from the onset to admission was 6 (IQR: 3–8.5) days.

**Table 1:** Demographics and clinical characteristics of 245 patients with COVID-19.

	**Total (*n* = 245)**	**Non-survivor (*n* = 23)**	**Survivor (*n* = 222)**	***P*-value**
Age, median (IQR)	54.0 (37.0–64.0)	76.0 (64.5–81.0)	52.0 (36.0–62.0)	<0.001
Gender				0.002
Female	127 (51.8%)	5 (21.7%)	122 (55.0%)	
Male	118 (48.2%)	18 (78.3%)	100 (45.0%)	
Any comorbidity	122 (49.8%)	19 (82.6%)	103 (46.4%)	0.001
Hypertension	72 (29.4%)	14 (60.9%)	58 (26.1%)	<0.001
Diabetes	31 (12.7%)	6 (26.1%)	25 (11.3%)	0.088
Chronic obstructive lung disease	7 (2.9%)	2 (8.7%)	5 (2.3%)	0.133
Cardiovascular disease	29 (11.8%)	9 (39.1%)	20 (9.0%)	<0.001
Carcinoma	11 (4.5%)	3 (13.0%)	8 (3.6%)	0.121
Nervous system disease	13 (5.3%)	5 (21.7%)	8 (3.6%)	0.001
Hepatic disease	19 (7.8%)	3 (13.0%)	16 (7.2%)	0.557
Respiratory rate>24 breaths per min	39 (15.9%)	5 (21.7%)	34 (15.3%)	0.616
**Initial symptoms**				
Fever	201 (82.0%)	16 (69.6%)	185 (83.3%)	0.176
Fatigue	132 (53.9%)	10 (43.5%)	122 (55.0%)	0.293
Cough	142 (58.0%)	11 (47.8%)	131 (59.0%)	0.301
Expectoration	70 (28.6%)	5 (21.7%)	65 (29.3%)	0.446
Dyspnea	70 (28.6%)	8 (34.8%)	62 (27.9%)	0.489
Muscle ache	56 (22.9%)	3 (13.0%)	53 (23.9%)	0.239
Headache	19 (7.8%)	2 (8.7%)	17 (7.7%)	1.000
Sore throat	18 (7.3%)	1 (4.3%)	17 (7.7%)	0.873
Diarrhea	27 (11.0%)	1 (4.3%)	26 (11.7%)	0.469
Rhinorrhea	7 (2.9%)	2 (8.7%)	5 (2.3%)	0.133
Onset to admission (IQR)	6.00 (3.00–8.50)	4.00 (1.00–7.50)	6.00 (3.75–9.00)	0.025

*IQR, interquartile range*.

### Laboratory Results and Treatments

Table 2 summarizes the laboratory results of the study population. On admission, 168 (73.0%) patients had lymphocyte counts below the normal range, while 60 (26.0%) of them had it within the normal range. The non-survivor group had higher counts of white blood cells (median value: 6.88 vs. 4.19) and neutrophils (5.83 vs. 2.60) compared to the survivor group while the latter had lower counts of lymphocytes (0.85 vs. 0.91) and platelets (157.00 vs. 166.50). Compared to the survivor group, the non-survivor group had three times higher levels of D-dimer as well as higher baseline levels of alanine aminotransferase (ALT), aspartate transaminase (AST), total bilirubin, serum creatine, all infection-related biomarkers and brain natriuretic peptide. Based on chest x-rays and CT manifestations, 91.9% (159/173) of the patients had pneumonia performance. Among them, 136 patients had bilateral pneumonia and 23 patients had unilateral pneumonia. Table 3 shows information on treatment, medication, and clinical outcomes. In all the cases, more than 50% of the patients received antiviral treatment, 73.9% of the patients were given synthetic corticosteroids (methylprednisolone/dexamethasone), and 9% (22/245) of the patients had respiratory failure. Among the 80 cases with ongoing viral nucleic acid testing data, 95% (76/80) of them tested negative within 14 days from onset (continuous bilateral detection) and there was one death case. In all the other non-survivors, the virus was detectable until death. The imaging results showed that in 16% (18/112) of the patients, the disease was in the progressive state. Of these, 87.5 and 10.6% were in the non-survivor and survivor groups, respectively.

**Table 2:** Laboratory results of 245 patients with COVID-19.

	**Median (IQR)/number (%)**				
	**Normal range**	**Total**	**Non-survivor**	**Survivor**	***P*-value**
**Blood routine**					
Leucocytes (× 10^9^/L)	3.5–9.5	4.30 (3.01–6.00)	6.88 (3.13–10.98)	4.19 (3.01–5.55)	0.015
<3.5		80/229 (34.9%)	6/20 (30.0%)	74/209 (35.4%)	0.106
3.5–9.5		127/229 (55.5%)	8/20 (40.0%)	119/209 (56.9%)	
>9.5		22/229 (9.6%)	6/20 (30.0%)	16/209 (7.7%)	
Neutrophils (× 10^9^/L)	1.8–6.3	2.91 (1.88–4.95)	5.83 (3.65–9.57)	2.60 (1.82–4.29)	0.001
Lymphocytes (× 10^9^/L)	1.1–3.2	0.91 (0.66–1.11)	0.85 (0.56–1.07)	0.91 (0.66–1.11)	0.647
<1.1		168/230 (73.0%)	17/22 (77.3%)	151/208 (72.6%)	0.827
1.1–3.2		60/230 (26.0%)	3/22 (13.6%)	57/208 (27.4%)	
>3.2		2/230 (1.0%)	2/22 (9.1%)	0	
Platelets (× 10^9^/L)	125.0–350.0	166.00 (124.75–200.00)	157.00 (112.25–187.75)	166.50 (126.00–202.50)	0.327
<125.0		55/220 (25.0%)	7/20 (35.0%)	48/200 (24.0%)	0.450
125.0–350.0		162/220 (74.0%)	12/20 (60.0%)	150/200 (75.0%)	
>350.0		3/220 (1.0%)	1/20 (5.0%)	2/200 (1.0%)	
Hemoglobin (g/L)	130.0–175.0	131.00 (118.00-141.00)	127.50 (93.95–136.48)	131.00 (119.00–142.00)	0.111
**Coagulation function**					
Prothrombin time (s)	9.4–12.5	12.15 (11.28–13.10)	12.10 (11.40–13.10)	12.20 (11.25–13.05)	0.640
Activated partial thromboplastin time (s)	25.1–36.5	30.20 (28.00–33.00)	28.85 (26.50–34.50)	30.25 (28.00–32.70)	0.853
D-dimer (ng/L)	0–500	240.00 (108.00–633.00)	762.00 (10.18–1574.75)	230.00 (108.50–505.00)	0.079
**Blood biochemistry**					
Albumin (g/L)	40–55	38.20 (34.90–41.48)	34.55 (32.73–38.48)	38.60 (35.38–41.60)	0.004
Alanine aminotransferase (U/L)	9–50	25.00 (15.45–42.23)	30.20 (18.90–55.00)	25.00 (15.30–39.00)	0.267
Aspartate aminotransferase (U/L)	15–40	27.55 (19.30–42.78)	34.00 (25.00–54.00)	27.00 (19.30–40.00)	0.179
Total bilirubin (μmol/L)	5.0–21.0	9.40 (7.60–13.22)	15.50 (7.90–22.05)	9.30 (7.60–12.50)	0.058
Potassium (mmol/L)	3.5–5.3	4.02 (3.61–4.36)	4.13 (3.91–4.94)	4.01 (3.60–4.35)	0.116
Sodium (mmol/L)	137.0–147.0	139.40 (135.90–141.60)	139.30 (132.00–141.00)	139.45 (136.00–141.60)	0.598
Serum creatinine (μmol/L)	64.0–104.0	67.90 (54.40–84.40)	96.60 (72.90–133.25)	67.15 (53.30–81.55)	0.001
**Myocardial enzyme**					
Myoglobin (ng/mL)	<140.1	50.20 (31.20–106.30)	158.60 (84.08–245.95)	48.30 (25.75–72.50)	0.001
**Infection-related biomarkers**					
Procalcitonin (ng/mL)	<0.05	0.09 (0.06–0.22)	0.23 (0.13–1.07)	0.08 (0.06–0.15)	<0.001
<0.05		118/220 (53.6%)	3/22 (13.6%)	115/198 (58.1%)	<0.001
≥0.05		102/220 (46.4%)	19/22 (86.4%)	83/198 (41.9%)	
Interleukin-6 (pg/mL)	0–7.0	22.81 (6.94–58.66)	68.59 (32.64–223.23)	18.47 (5.68–45.03)	0.001
<7.0		32/118 (27.1%)	2/16 (12.5%)	30/102 (29.4%)	0.159
≥7.0		86/118 (72.9%)	14/16 (87.5%)	72/102 (70.6%)	
Erythrocyte sedimentation rate (mm/h)	0–20	25.00 (13.25–53.00)	80.00 (48.00–96.00)	24.00 (13.00–43.00)	<0.001
<20		57/141 (40.4%)	2/13 (15.4%)	55/128 (43.0%)	0.054
≥20		84/141 (59.6%)	11/13 (84.6%)	73/128 (57.0%)	
C-reactive protein (mg/L)	0–10	5.90 (1.82–25.05)	9.46 (3.27–68.94)	5.35 (1.80–24.32)	0.106
<10		120/201 (59.7%)	10/19 (52.6%)	110/182 (60.4%)	0.510
≥10		81/201 (40.3%)	9/19 (47.4%)	72/182 (39.6%)	
Brain natriuretic peptide (pg/mL)	0–1,800.0	29.70 (10.00–189.85)	283.80 (10.20–363.00)	24.23 (10.00–107.55)	0.013
**Chest x-ray and CT manifestations**					
Bilateral pneumonia		136/173 (78.6%)	14 (87.5%)	122/157 (77.7%)	0.235
Unilateral pneumonia		23/173 (13.3%)	2 (12.5%)	21/157 (13.4%)	
No abnormality		14/173 (8.1%)	0	14/157 (8.9%)	

**Table 3:** Clinical treatments and outcomes of patients with COVID-19.

**Project**	**Total**	**Non-survivor**	**Survivor**	***P*-value**
	**(*n* = 245)**	**(*n* = 23)**	**(*n* = 222)**	
Antibiotic therapy	238 (97.1%)	22 (95.7%)	216 (97.3%)	0.503
**Antiviral therapy**				
Oseltamivir	131 (53.5%)	14 (60.9%)	117 (52.7%)	0.455
Ribavirin	43 (17.6%)	5 (21.7%)	38 (17.1%)	0.79
Abidol	64 (26.1%)	4 (17.4%)	60 (27.0%)	0.317
Alpha-interferon	19 (7.8%)	1 (4.3%)	18 (8.1%)	0.490
Corticosteroid treatment	181 (73.9%)	20 (87.0%)	161 (72.5%)	0.134
Respiratory failure	22 (9.0%)	9 (39.1%)	13 (5.9%)	<0.001
**Onset to RT-PCR turning negative (d)**				
≤14	76/80 (95.0%)	1	75 (94.9%)	0.819
>14	4/80 (5.0%)	0	4 (5.1%)	
**Imaging changes**				
Improvement	88/112 (78.6%)	0	88 (84.6%)	<0.001
Progression	18/112 (16.0%)	7/8 (87.5%)	11 (10.6%)	
No change	6/112 (5.4%)	1 (12.5%)	5 (4.8%)	

### The Risk of Death

The univariable analysis found higher odds of in-hospital death in patients with chronic obstructive lung disease, and CVD (Table 4). Age, sex, lymphocyte counts, prothrombin time, levels of albumin, ALT, sodium, myoglobin, C-reactive protein, and ribavirin were also significantly associated with death (*p* < 0.05) (Table 4). The multivariate regression analysis showed (Table 5) that the risk of hospitalization death increased with age [hazard ratio (HR): 1.165, 95% CI (confidence interval): 1.051–1.292, *p* = 0.004], D-dimer >1,000 ng/L (HR: 11.516, 95% CI: 1.157–114.658, *p* = 0.037), platelets <125 × 10^9^/L (HR: 7.731, 95% CI: 1.303–45.674, *p* = 0.024), and higher serum creatinine levels (HR: 1.004, 95% CI: 1.002–1.007, *p* = 0.002). However, cardiovascular disease and lymphocyte counts showed no significant association with death based on the multivariate analysis.

**Table 4:** Univariate cox regression analysis of clinical parameters in patients with COVID-19.

	**Univariable OR (95% CI)**	***P*-value**
**Demographics and clinical characteristics**		
Age, years	1.089 (1.049–1.132)	<0.001^*^
Male sex (vs. female)	3.024 (1.110–8.237)	0.030^*^
Comorbidity present (vs. not present)	2.667 (0.891–7.982)	0.079
Chronic obstructive lung disease	5.050 (1.115–22.867)	0.036^*^
Hypertension	1.966 (0.829–4.664)	0.125
Diabetes	1.404 (0.535–3.683)	0.490
Cardiovascular disease	2.637 (1.093–6.360)	0.031^*^
Carcinoma	3.252 (0.941–11.240)	0.062
Nervous system disease	1.668 (0.571–4.878)	0.350
Hepatic disease	1.478 (0.435–5.027)	0.531
**Blood routine**		
**Leucocytes (× 10^9^/L)**		
<3.5	1.315 (0.453–3.820)	0.614
3.5–9.5	Ref	
>9.5	2.493 (0.833–7.457)	0.102
Neutrophils (× 10^9^/L)	1.116 (0.985–1.264)	0.085
**Lymphocytes (× 10^9^/L)**		
<1.1	2.432 (0.692–8.543)	0.166
1.1–3.2	Ref	
>3.2	22.499 (3.434–147.387)	0.001^*^
**Platelets (× 10^9^/L)**		
<125.0	1.863 (0.720–4.821)	0.199
125.0–350.0	Ref	
>350.0	2.337 (0.291–18.755)	0.424
**Hemoglobin (g/L)**		
**Coagulation function**		
Prothrombin time (s)	1.791 (1.348–2.379)	<0.001^*^
Activated partial thromboplastin time (s)	1.008 (0.998–1.019)	0.115
D-dimer (ng/L)	1.000 (1.000–1.000)	0.130
**Blood biochemistry**		
Albumin (g/L)	0.924 (0.858–0.996)	0.038^*^
Alanine aminotransferase (U/L)	1.002 (1.000–1.003)	0.009^*^
Aspartate aminotransferase (U/L)	1.001 (0.998–1.004)	0.580
Total bilirubin (μmol/L)	0.999 (0.963–1.037)	0.976
Potassium (mmol/L)	1.454 (0.813–2.602)	0.207
Sodium (mmol/L)	0.971 (0.943–0.999)	0.045^*^
Serum creatinine (μmol/L)	1.001 (1.000–1.002)	0.089
**Myocardial enzyme**		
Myoglobin (ng/mL)	1.005 (1.002–1.007)	<0.001^*^
**Infection-related biomarkers**		
**Procalcitonin (ng/mL)**		
<0.05	Ref	
≥0.05	6.090 (0.806–46.016)	0.080
**Interleukin-6 (pg/mL)**		
<7.0	Ref	
≥7.0	3.505 (0.778–15.787)	0.102
**Erythrocyte sedimentation rate (mm/h)**		
<20	Ref	
≥20	3.666 (0.461–29.140)	0.219
**C-reactive protein (mg/L)**		
<10	Ref	
≥10	4.140 (1.583–10.829)	0.004^*^
**Brain natriuretic peptide (pg/mL)**		
**Clinical treatments**		
Oseltamivir	1.867 (0.791–4.405)	0.154
Ribavirin	0.275 (0.088–0.862)	0.027^*^
Abidol	0.364 (0.107–1.245)	0.107
Corticosteroid treatment	0.426 (0.111–1.641)	0.215
Alpha-interferon	0.434 (0.054–3.497)	0.433
**Multicenter**		
Zhongnan hospital	Ref	
Central hospital	0.369 (0.151–0.901)	0.029^*^

* *P < 0.05*.

**Table 5:** Multivariate cox regression analysis of clinical parameters in patients with COVID-19.

	**Multivariable OR (95% CI)**	***P*-value**
Age	1.165 (1.051–1.292)	0.004^*^
Cardiovascular disease	0.312 (0.066–1.483)	0.143
**Lymphocytes (× 10^9^/L)**		
<1.1	5.387 (0.810–35.848)	0.082
1.1–3.2	Ref	
**Platelets (× 10^9^/L)**		
<125	7.731 (1.303–45.674)	0.024^*^
≥125	Ref	
**D-dimer (ng/L)**		
<500	Ref	
500–1,000	3.601 (0.308–42.089)	0.307
>1,000	11.516 (1.157–114.658)	0.037^*^
Serum creatinine	1.004 (1.002–1.007)	0.002^*^
Total bilirubin	1.037 (0.980–1.098)	0.209
Respiratory failure	2.456 (0.645–9.361)	0.188

* *P < 0.05*.

## Discussion

Though the epidemiology of patients with COVID-19 is widely studied and reported, the death-related risk factors and detailed clinical characteristics of the disease have not been well-described. In this study, we summarized the clinical characteristics of 245 patients diagnosed with COVID-19 at two centers in Wuhan. We also identified risk factors associated with COVID-19-related deaths. These included older age, D-dimer > 1,000 ng/L, platelets < 125 × 10^9^/L, and higher serum creatinine levels.

In our study, 9.4% of the patients died of COVID-19. While the average age of the total cohort was 54 years, that of the non-survivor group was 76 years. In another retrospective, multicenter study on COVID-19 cases from Wuhan (9), 54 (28.3%) of the patients died in hospital, the median age of 191 patients was 56 (IQR 46–67) years, and that of the non-survivors was 69 (63–76) years, which was comparable to our findings. In a study by Zhong et al. (10), which enrolled 1,099 laboratory confirmed COVID-19 cases from 552 hospitals across China, the median age of the study population was 47 years and 1.4% of the patients died of the disease. The fatality rate in Wuhan is significantly higher than that in other regions. It is probably because of the concentration of a large number of cases.

It has been reported that older patients have a worse prognosis of the Middle East respiratory syndrome (MERS) and severe acute respiratory syndrome (SARS) (11, 12). Previous studies have found that older age is related to stronger host innate responses to virus infection and stronger activation of the pro-inflammatory pathways, with a marked decline in cell-mediated as well as humoral immune functions (13, 14).

In addition to age factors, we also found D-dimer levels >1,000 ng/L, and platelet counts < 125 × 10^9^/L on admission were associated with fatal outcomes in COVID-19 patients. High levels of D-dimer have been verified to be a significant prognostic factor in patients with suspected infection and sepsis (15). Increased D-dimer is distinctly associated to disseminated intravascular coagulation, which is considered as an early stage of diffuse pulmonary intravascular coagulopathy (16). Extensive thrombosis in small vessels and the microvasculature has been confirmed histologically as one of the important basic pathological changes of lung in hospitalized COVID-19 patients (17). Platelets also play an equally important role as a consumable substance in the clotting process. Therefore, the reduction of platelets may also affect the survival status of patients through the same way. As we all know, COVID-19 has many similarities to SARS. In a retrospective study, 20–45% of SARS patients had thrombocytopenia (18). There are 7(35%) of non-survivor patients had platelets counts below the normal range on admission. Moreover, severe infections could lead to secondary thrombocytopenia characterized by a rapid decline in platelet count (19). These conditions are associated with damages to the hematopoietic system and the lungs, in which mature megakaryocytes release platelets (20). However, Qu et al. (21) have reported that while COVID-19 patients with significantly elevated platelet count during treatment were on an average hospitalized longer, the platelet count at admission was lower in severely ill patients (169.67 ± 48.95) compared to those who were not severely ill (192.26 ± 58.12). The initial increase in platelet count, followed by a decrease in severely ill patients, may be associated with the progression of COVID-19.

Basal CVD did not show a significant association with COVID-19-related mortality. Coronary heart disease has been correlated with acute cardiac incidents and adverse outcomes in respiratory viral infections (22–24). However, consistent with our findings, previous studies (9) have also verified that CVD did not play an independent role in multivariate regression analysis of COVID-19-related mortality.

This study has some limitations. First, laboratory tests such as hypersensitive troponin, creatine kinase, and serum ferritin were missing because the study was retrospective. At the same time, because many cases were hospitalized in the early stage of the epidemic, there are also many missing data of viral nucleic acid test. Second, the outcomes were evaluated at the end of the follow-up period instead of at a fixed time point during the course of the disease. Finally, the sample size was small. Hence, the interpretation of our results might be limited. However, by including patients from two appointed hospitals for COVID-19, we believe the object of our study is representative of the total patient population.

Although Wuhan achieved victory over COVID-19 through hard work, this new coronary pneumonia is now a worldwide disease. The virus can be eliminated only if all countries take appropriate measures. The medical workers in Wuhan have accumulated experience fighting this epidemic, and have no reservations in providing advice to colleagues in need. We are also committed to passing on our knowledge and expertise to other countries currently fighting COVID-19.

## Conclusions

In conclusion, almost 9.4% of our study population died of COVID-19. Older age, elevated D-dimer levels, and thrombocytopenia on admission were independent risk factors for death. More studies on the risk factors associated with COVID-19-related death are needed to control disease progression and to improve its treatment.

## Data Availability Statement

The raw data supporting the conclusions of this article will be made available by the authors, without undue reservation.

## Ethics Statement

The studies involving human participants were reviewed and approved by Zhongnan Hospital of Wuhan University (NO. 2020015) and the Central Hospital of Wuhan (NO. 2020072). Written informed consent for participation was not required for this study in accordance with the national legislation and the institutional requirements.

## Author Contributions

LG, ZL, and BC: conceptualization. SW, YH, WL, SC, ML, WZh, DC, LZ, and MWa: data collection and processing. BC, MT, and HX: interpretation. All authors: wrote-review and editing.

## Conflict of Interest

The authors declare that the research was conducted in the absence of any commercial or financial relationships that could be construed as a potential conflict of interest.
